# The Use of Synthetic Carriers in Malaria Vaccine Design

**DOI:** 10.3390/vaccines3040894

**Published:** 2015-10-29

**Authors:** Liam Powles, Sue D. Xiang, Cordelia Selomulya, Magdalena Plebanski

**Affiliations:** 1 Department of Chemical Engineering, Monash University, Clayton, VIC 3800, Australia; E-Mail: liam.powles@monash.edu; 2 Department of Immunology and Pathology, Monash University, Melbourne, VIC 3004, Australia; E-Mail: sue.xiang@monash.edu; 3 Therapeutics and Regenerative Medicine Division, The Monash Institute of Medical Engineering (MIME), Monash University, Clayton, VIC 3800, Australia

**Keywords:** malaria, vaccine, vector, synthetic, properties, particles, nanoparticles

## Abstract

Malaria vaccine research has been ongoing since the 1980s with limited success. However, recent improvements in our understanding of the immune responses required to combat each stage of infection will allow for intelligent design of both antigens and their associated delivery vaccine vehicles/vectors. Synthetic carriers (also known as vectors) are usually particulate and have multiple properties, which can be varied to control how an associated vaccine interacts with the host, and consequently how the immune response develops. This review comprehensively analyzes both historical and recent studies in which synthetic carriers are used to deliver malaria vaccines. Furthermore, the requirements for a synthetic carrier, such as size, charge, and surface chemistry are reviewed in order to understand the design of effective particle-based vaccines against malaria, as well as providing general insights. Synthetic carriers have the ability to alter and direct the immune response, and a better control of particle properties will facilitate improved vaccine design in the near future.

## 1. Introduction

The World Health Organization estimates that in 2013 there were 198 million cases of malaria; 584,000 of which were fatal [1]. The disease is prevalent in many tropical and subtropical regions of the world, most notably Sub-Saharan Africa, Asia, Latin America and the Middle East [2]. Africa carries the majority of the burden of lethal infections, which are most often mediated by the *Plasmodium falciparum* parasite strains. Vaccination preventing infection and subsequent transmission is the most likely strategy to reduce the spread and resurgence of the disease. Research into malaria vaccine candidates has been ongoing for many years, with limited progress. This is largely due to the complexity of the parasites’ life cycle, the lack of complete understanding of the body’s natural protective immunity to infection, and the genetic polymorphism of potential antigenic targets [3,4]. The strongest candidate, the recombinant circumsporozoite protein (CSP) based pre-erythrocytic stage RTS,S vaccine (Mosquirix^TM^), has undergone phase III clinical trials and is likely to be licensed in 2015. However, efficacy results have varied from 30% to 50% in reduction rate of clinical episodes of malaria, with short lived and reduced protection in infants [5]. Therefore, further work is needed to develop a long lasting, efficacious vaccine against malaria. New generation attenuated whole parasite vaccines, recombinant technology, and nanotechnology are being actively explored to deliver such a vaccine [6,7]. Strongly immunogenic antigens with limited polymorphism are being discovered using immunomic techniques that allow for the analysis of many proteins identified in genomic data [8]. It is expected that future vaccine formulations will incorporate antigens against multiple stages of the malaria life cycle with improved adjuvants that are able to induce the desired types of protective immune responses.

Adjuvants can be defined as “any substance which when incorporated into a vaccine formulation acts generally to accelerate, prolong or enhance the quality of specific immune responses to vaccine antigens” [9]. Often the adjuvant effect relies on “danger signals” promoting the induction of inflammatory pathways [10]. Conversely, vaccine carriers or vectors (which can also have intrinsic adjuvant properties) are non-specific delivery systems to which recombinant protein, peptide or DNA antigens can be associated to promote their delivery to the antigen presenting cells (APCs) that then initiate an immune response [7]. Numerous carrier/vector characteristics such as size, shape, and surface charge, can be varied to alter the way the associated antigen is seen and processed by APCs and generally interacts with the immune system. This can in turn alter the strength and type of immune response. Furthermore, these properties allow for directing the immune response in a cellular or humoral direction, a practice considered especially important in malaria vaccine design [11].

Vectors historically used in clinical trials are based on attenuated organisms such as genetically altered viruses [5]. Diversification in recent years has seen the development of a wide range of synthetic particles including organic carriers such as liposomes and polymeric microspheres, and inorganic particles based on materials including iron oxide and calcium phosphate [12,13,14,15]. This review will focus on the requirements for a synthetic vaccine vector, both in general and for malaria specifically. It will then examine the properties that make micro- and nanoparticles attractive as vectors, and summarize the literature in which malaria vaccines are delivered with non-conventional vectors with respect to the aforementioned requirements.

## 2. Vaccine Carrier/Vector Requirements

Historically, successful vaccines have been developed using attenuated and killed organisms. For example, measles and mumps are still prevented using attenuated living viruses while polio vaccines based on both attenuated and inactivated poliovirus have been successful [16,17,18]. Whilst these vaccines have led to a vast reduction in the prevalence of disease, there are those for which progress in vaccine development has been limited, most notably HIV and malaria. In recent years, vaccine research has broadened with subunit and DNA vaccines seeing expanded development at the expense of attenuated and inactivated organism based approaches. Subunit vaccines contain antigens (peptide or protein) from the organism that are the target of specific immune responses [19]. DNA vaccines use genetic material that encodes for the antigens of interest. This material is internalized by cells in the body, which will then process and express the encoded protein. The displayed protein induces dendritic cell (DC) activation and subsequent adaptive immune responses [20]. The major advantages of subunit and DNA vaccines are their inherent safety, the ability to tailor their immune responses, simpler storage and transport requirements and easier development/production [21]. The main drawback of subunit vaccines is their weak immunogenicity, often necessitating advanced delivery systems and/or adjuvant use [22]. Similarly, naked DNA vaccines are quickly removed by the body and hence require delivery vectors. In addition, the immunogenicity evident in mice is often not maintained in humans when the DNA vaccine is tested clinically [23]. This has led to a focus on better vectors for improved cell transfection. Looking towards the future, it seems highly likely that subunit and DNA vaccines will continue to dominate research efforts. Thus, it is important that vectors are developed concurrently to improve their efficacy in a safe, affordable manner.

A successful vector will need to meet a number of requirements for clinical use. It should be designed to enhance and complement the natural response to the vaccine. In addition, vaccine formulations should be stable for extended periods of time and amenable to transport and storage in ambient conditions which are vital in underdeveloped regions [24]. For the same reason, it would be preferable if vaccines could be delivered via a single injection, without the need for secondary and tertiary administration. Production of vaccine vectors needs to be affordable, robust, and scalable. Furthermore, the need to manufacture multiple components (vaccine and vector) will increase costs compared with existing systems [25]. Lastly, and most importantly, a vector needs to be safe for human use, in terms of toxicity, acute and chronic side effects, biocompatibility, and biodegradability, especially for nanoparticle use in vaccines [26]. For particles in particular, size and surface reactivity can allow for diffusion to potentially susceptible areas such as the lungs and brain. Toxicity studies should be undertaken at the preclinical stage and *in vivo* distribution needs to be mapped.

## 3. Malaria Vaccine Requirements

**Figure 1:**
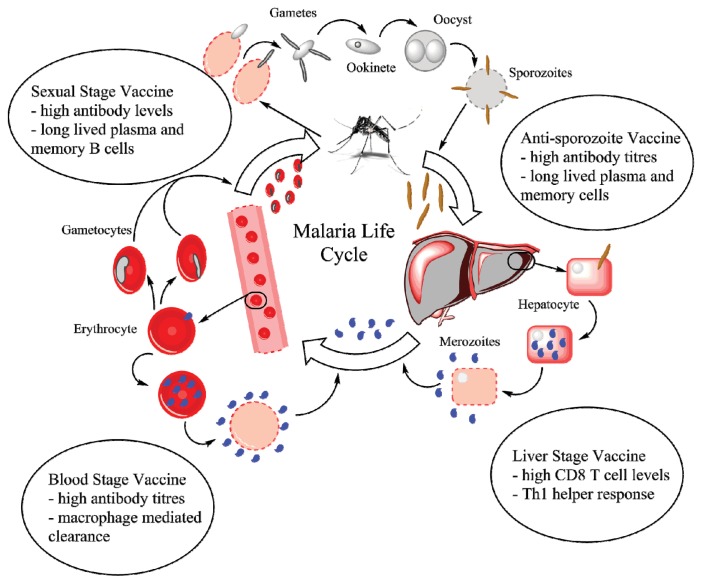
The life cycle of the malaria parasite. A mosquito delivers sporozoites into the blood stream, which quickly travel to and infect hepatocytes. Inside liver cells, the sporozoites differentiate and proliferate to form merozoites, which upon release infect erythrocytes. Proliferation and release continues inside infected erythrocytes with a small fraction of merozoites differentiating into gametocytes. The gametocyte-infected erythrocytes are taken up by mosquitoes inside which gametes interact to form a zygote (ookinete) and eventually an oocyst, which produces sporozoites. The bubbles contain the primary immune response required against each stage. Figure developed using ChemBioDraw Ultra 14 (PerkinElmer Informatics, Waltham, MA, USA, 2014).

The malaria parasite interacts with and affects its host in a distinct manner at each stage of its life cycle (Figure 1). Thus, in order to target each stage of infection (*i.e.*, prevention of cell invasion *versus* elimination of infected cells), different strategies are required. The problem is exacerbated in that each stage expresses different surface proteins requiring specific responses from the vaccine. The goal of any pre-erythrocytic vaccine is to prevent parasites exiting the liver; for example anti-sporozoite vaccines stop sporozoites infecting the liver [27]. A vaccine targeting sporozoites needs to induce high antibody titers with strong avidity to surface antigens to either prevent the parasite leaving the injection site, or inhibit liver infection [28]. In addition, long lived plasma cells and memory cells may be required to maintain immunity over a long period of time [29]. The molecular mechanisms of hepatocyte invasion are unknown, though a number of proteins thought to be important to the process have been investigated. The CSP is the most abundant surface protein and has been implicated in motility and cell invasion [30,31]. Other identified targets for pre-erythrocytic candidate vaccines include thrombospondin-related anonymous protein (TRAP) which plays roles in motility and invasion, apical membrane antigen 1 (AMA1) involved in hepatocyte invasion and cell traversal protein (CelTOS), and sporozoite microneme protein essential for cell traversal (SPECT), part of the cell traversal mechanics [32,33,34,35,36]. As well as blocking hepatocyte invasion, pre-erythrocytic stage vaccines can also be designed to prevent merozoites exiting into the blood stream by inhibiting parasite maturation inside the liver cells [28]. Activation of sporozoite-specific CD8+ T cells promotes their expansion, cytotoxicity, and cytokine secretion (importantly IFN-γ and TNF-α) [37,38]. These cells then kill infected cells exhibiting the antigen their ancestors initially responded against. Sporozoite antigens such as CSP and TRAP are still exhibited after hepatocyte invasion, along with liver-stage antigens, which can be targeted by vaccines. These include liver-stage antigen 1 (LSA1), involved in liver-stage differentiation, liver-stage antigen 3 (LSA3), and exported protein 1 (Exp1), a membrane protein [39,40]. The use of antigens expressed only in the liver stage is attractive as they are often highly conserved [40,41]. Extremely high cytotoxic T cell responses are needed to combat liver-stage infection. Therefore, a vaccine system needs to target DCs capable of cross-priming into MHC class I pathways and elicit high levels of CD8+ T cells, as well as a supporting CD4+ helper T cell type 1 (Th1) response, which generates the high levels of IL-2 production necessary to sustain CD8+ T cell expansion [42,43].

Blood stage vaccines aim to inhibit erythrocyte invasion and/or parasite reproduction, as well as opsonize infected cells for macrophage removal [28]. A vaccine that meets these requirements will induce high antibody titers with a high affinity for their substrate, cytokine production for phagocyte activation and generation of long-lived plasma and B and T memory cells. Evidence suggests that high antibody titers may be required for protection, and that in mice, natural killer (NK) cells as well as B and T cells play important roles [44,45,46]. Specifically NK and T cell derived IFN-γ promote efficient splenic macrophage activation, and hence parasite clearance. Most individuals living in malaria endemic regions show antibody responses to a large number of Plasmodium blood stage proteins [47]. While this provides potential antigenic targets for vaccines, the pressure placed on the parasite has led to a high degree of allelic variation [48,49]. In many cases this reduces vaccine efficacy throughout a population and promotes further polymorphic immunity to the vaccine. The antigens commonly investigated include merozoite surface protein 1 (MSP1) which is likely to be involved in erythrocyte attachment, merozoite surface protein 2 (MSP2) and merozoite surface protein 3 (MSP3) whose functions are unknown but thought to be essential, and AMA1 and erythrocyte binding antigen 175 (EBA175) which are involved in blood cell invasion [50,51,52,53]. Recent work has focused on *Plasmodium falciparum* reticulocyte binding-like homologous protein 5 (PfRH5) due to its highly conserved nature [54]. This is exciting as the antigen is thought to play an essential role in erythrocyte invasion [55]. 

The goal of sexual stage vaccines (transmission blocking vaccines) is to prevent gametocyte uptake by the mosquito and/or to inhibit parasite development within the vector [28]. This would be solely antibody based, with gametocyte, gamete or ookinete antigens targeted to inhibit fertilization or migration. In addition, antibodies can mediate gamete lysis through the human complement system [56]. For antigen which is only expressed on the parasite surface during mosquito inhabitation, long-lived plasma cells and memory B cells would be especially important, as the lack of re-exposure would prevent natural boosting of the response upon infection [57]. A sexual stage vaccine would present no benefit to the vaccinated person and would instead reduce the spread of malaria over the whole population. The most commonly investigated antigens are *Plasmodium falciparum* surface protein 25 (Pfs25) and *Plasmodium vivax* surface protein 25 (Pvs25). These proteins are expressed on the surface of the parasites zygote and ookinete stages [58]. Other antigens being investigated include *Plasmodium vivax* surface protein 230 (Pvs230), *Plasmodium falciparum* surface protein 230 (Pfs230), and *Plasmodium falciparum* surface proteins P48/45 (Pfs48/45). These are expressed by the gametocyte and gamete stages and can therefore rely on natural immune boosting [59,60,61]. Vectors for this stage have been suggested to need to promote strong T follicular helper (T_fh_) and Th2 responses.

## 4. Particle Use in Malaria Vaccine Development

**Figure 2:**
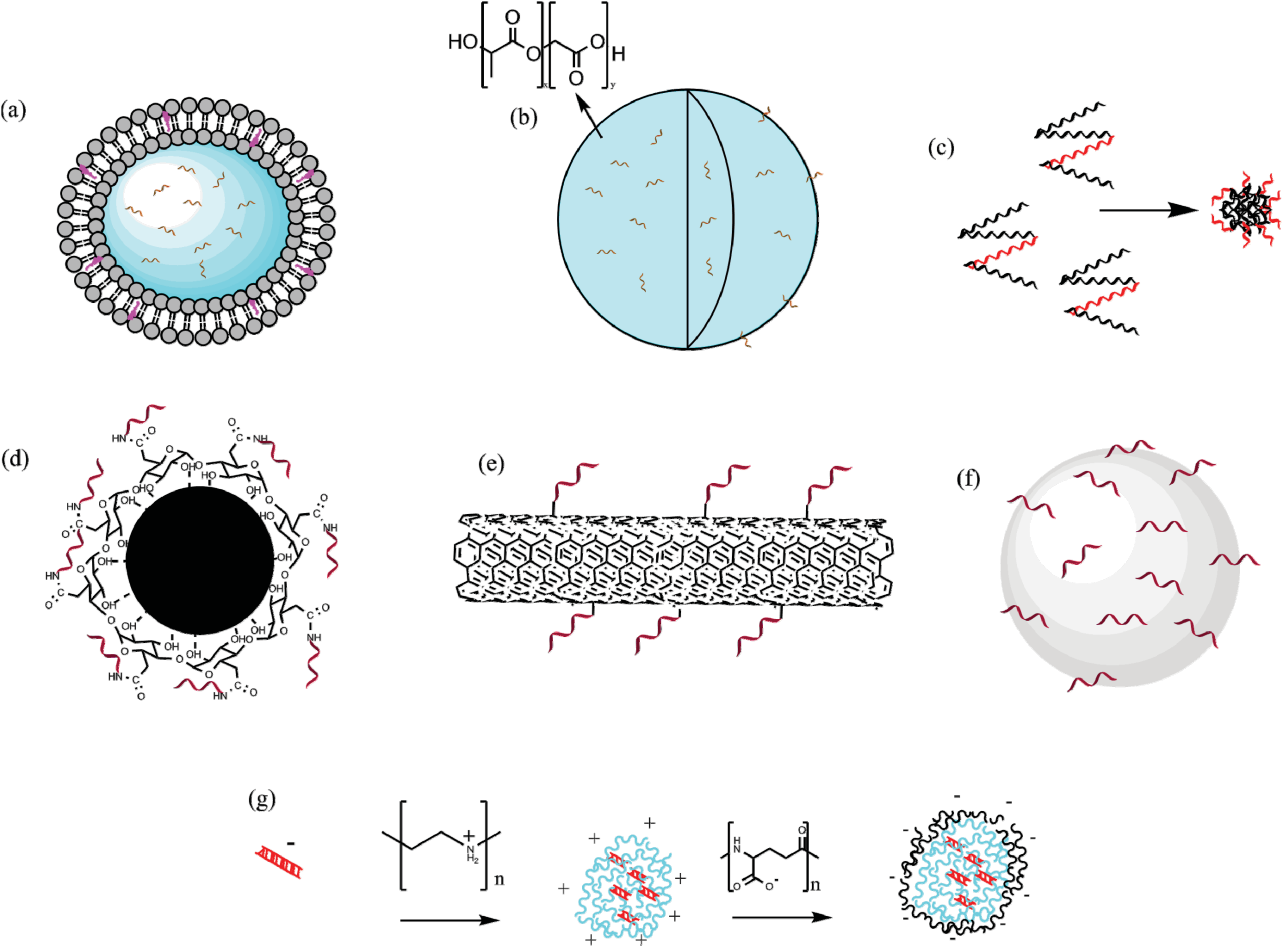
A selection of different particle types that have been used as vectors for malaria vaccines. Note the different vaccine types and methods of incorporation. (**a**) PC liposomes, the antigen is encapsulated inside an aqueous compartment; (**b**) PLGA particles, antigen encapsulated both in and on the surface of particle; (**c**) Self assembled protein nanoparticles, peptide incorporated into protein to enable repetitive display; (**d**) Iron oxide nanoparticle, antigen covalently conjugated to polymeric coating; (**e**) Carbon Nanotube; (**f**) Hydroxyapatite particle, antigen adsorbed to the surface; (**g**) PEI/PGA nanoparticle construct, DNA vaccine electrostatically bound to PEI. Figure developed using ChemBioDraw Ultra 14.

This section will highlight a number of historical and recent malaria studies in which a wide range of different materials have been used as particle-based vectors for recombinant protein, peptide, or DNA encoding malarial antigens (summarized in Table 1). Although we have attempted to be comprehensive, we apologize to the authors of papers that may have been inadvertently omitted. There is great variability in these studies in terms of antigenic targets, particle types (highlighted in Figure 2), particle properties, and study goals. However, together they provide useful specific insights into the state of this active and exciting field of research, which will be discussed by categorizing the studies into broad classes based on the type of immune response elicited, the malarial stage they target, and the particle type, to identify and highlight the most informative results.

**Table 1:** Summary of clinical and preclinical studies incorporating novel synthetic vector strategies.

Vector	Size	Vaccine	Antibodies	Cytotoxic Response	Ref.
**Pre-Erythrocytic Candidates**					
*Lipid Based Particles*					
PC ^1^ liposomes	Not reported	PyCSP ^2^ (tetrapeptide B cell, other B, T cell epitopes, RTS,S)	Yes	Some antigens	[12,62,63,64,65,66,67,68,69]
Lipid core peptides	Not reported	PyCSP (CD4 and CD8 epitopes)	Minor IgE	ND ^3^	[70]
ICMV ^4^	180 nm (DLS ^5^)	PvCSP ^6^ (VMP001)	Yes	ND	[71]
*Polymeric Particles*					
PCL ^7^/PLA ^8^	23–45 µm	PfCSP ^9^ (tetrapeptide, universal CD4)	Yes	ND	[72]
PLA/PLGA ^10^	1–100 µm	PfCSP (tetrapeptide, universal CD4), Pb9^11^	Yes	Against Pb9	[13,73,74]
PLGA	0.45–32.1 µm	Pb9	ND	Yes	[75]
Lipid enveloped PLGA	290 nm (DLS)	PvCSP (VMP001)	Yes	ND	[76]
Polystyrene Nanoparticles	48 nm (DLS)	Pb9	ND	Yes	[77]
*Other Particles*					
SAPN ^12^	40 nm (TEM ^13^, DLS)	PfCSP, PvCSP, CD8 and B epitopes, universal CD4	Yes	Some antigens	[78,79,80]
Polymer coated calcium carbonate	Not reported	PfCSP (B and T cell epitopes)	Yes	Yes	[81]
**Blood Stage Candidates**					
*Lipid Based Particles*					
PC Liposome	Not reported	PfMSP1–19 ^14^	Yes	ND	[82]
PC Liposome	Not reported	PyIMP-66 ^15^	Yes	ND	[83]
*E. coli* ^16^ Liposome	Not reported	Py soluble	Yes	Yes	[84]
*S. cer.* ^17^ Liposome	Not reported	Py soluble	Yes	Yes	[85]
pH sensitive Liposome	325–390 nm	PfMSP1–19	Minor	ND	[70]
*Other Organic Particles*					
PEI ^18^, γ-PGA ^19^	68 nm (DLS)	PyMSP1-C-terminus, PyTAM ^20^ (DNA)	Yes	With PyTAM	[86,87,88]
ISCOM ^21^	Not Reported	PfRESA ^22^	Yes	ND	[89,90]
ISCOM	Not Reported	PfRESA peptides	Yes	ND	[91]
*Inorganic Particles*					
SPIONs ^23^	20 nm (unknown)	PfMSP1–42	Yes	ND	[14]
PEI coated SPIONs	147 nm (DLS)	PyMSP1–19 (DNA)	Yes	ND	[92]
Gold nanoparticle	17 nm (TEM)	Pf/PvMSP1–19	Yes	ND	[93]
Quantum Dots	15 nm (unknown)	PfMSP1–42	Yes	ND	[94]
Carbon Nanotubes	20–30/500–2000 nm	PvAMA1 ^4^peptides	Yes	ND	[95]
Hydroxyapatite	784 nm (DLS)	MSP1–19	Yes	ND	[15]
**Mixed Stage Candidates**					
PLGA	0.5–2 µm	SPf66, PfMSP2 ^25^ peptides, PfS3	Yes	ND	[96,97,98,99,100,101,102]
PLGA-alginate-RGD	0.8–1 µm	SPf66, PfS3	Yes	ND	[103]
PLGA	1–2 µm	PvCSP, MSP1, AMA1, Pvs24 (all with B and T epitopes)	Yes	ND	[104,105]
**Sexual Stage Candidate**					
Gel core liposomes	1–1.2 µm	Pfs25	Yes	ND	[106]

^1^: Phosphatidylcholine; ^2^: *Plasmodium yoelii* circumsporozoite protein; ^3^: Not Done; ^4^: Interbilayer-crosslinked multilamellar vesicles; ^5^: Dynamic light scattering; ^6^: *Plasmodium vivax*; ^7^: Polycaprolactone; ^8^: Poly(lactic acid); ^9^: *Plasmodium falciparum*; ^10^: Poly(lactic-co-glycolic acid); ^11^: *Plasmodium berghei*; ^12^: Self-Assembling protein nanoparticle; ^13^: Transmission electron microscopy; ^14^: Merozoite Surface Protein 1; ^15^: Integral Membrane Protein; ^16^: *Escherichia coli*; ^17^: *Saccharomyces cerevisiae*; ^18^: Polyethyleneimine; ^19^: γ-polyglutamic acid; ^20^: PyGPI8p-transamidase-related protein; ^21^: Immune stimulating complex; ^22^: Ring-infected erythrocyte surface antigen; ^23^: Superparamagnetic iron oxide nanoparticles; ^24^: Apical Membrane Antigen 1; ^25^: Merozoite Surface Protein 2.

### 4.1. Pre-Erythrocytic Vaccines

Anti-sporozoite vaccines attempt to produce antibodies, which agglutinate or otherwise inactivate sporozoites such that they cannot infect hepatocytes. On the other hand, liver-stage vaccines require strong cytotoxic T cell responses to destroy infected cells [28]. Therefore, synthetic particle vector studies focused on meeting either one or both of these requirements are discussed below. A summary of the advantages and disadvantages of the different particle types used is given in Table 2.

**Table 2:** Comparison of advantages and disadvantages of different nanoparticle types.

Particle Type	Advantages	Disadvantages	Ref.
Lipid-Based	Wide size range Antigen encapsulated or on surface Hydrophobic or hydrophilic antigen FDA approved/Non-toxic Biodegradable	Expensive materials Reproducibility issues Oxidative Degradation	[107,108,109,110]
PLGA	Antigen encapsulated or on surface Biodegradable FDA approved/Non-toxic Prolonged release of antigen	Antigen degradation Scale-up Antigen burst releases	[110,111,112,113,114]
Polystyrene	Biocompatible Non-toxic Wide size range Readily available	Non-biodegradable	[115]
SAPN	Repetitive presentation Biodegradable	Complex design Limited Data	[116]
PEI/γ-PGA	Good for DNA vaccine Small size	Limited Data	[117]
ISCOM	Natural adjuvant Readily available Biodegradable Scalable Well-tolerated	Encapsulation limited Single size	[118,119,120]
SPION	Biodegradable Magnetic FDA approved Size control	Coating required Stability issues	[121,122]
Quantum Dot	Fluorescent Stable	Toxic materials Non-biodegradable	[123,124,125]
Calcium Based	Low cytotoxicity Surface modification	Limited degradability Limited study	[126,127]
Gold	Size control Low cytotoxicity	Non-biodegradable Coating required	[128,129]

#### 4.1.1. Lipid-Based Particles

Lipids are naturally occurring biological molecules whose properties can allow them to form structures in a suitable environment. Liposomes, the most of common of these, are vesicles formed when one or more lipid bilayers enclose an aqueous compartment [130]. Their use as drug delivery agents stretches back to the 1960s with research incorporating malaria antigens beginning in the 1980s [62,131]. Sizes range between 30 nm and several microns, and can be varied alongside lipid composition and surface charge to optimize their carrier potential [132]. For instance, incorporation of PEG into the lipid surface enhances the distribution time in blood [133]. Antigen is commonly encapsulated in the aqueous center of the liposome, with surface conjugation used to a lesser degree [134]. Advantages include the ability to incorporate both hydrophilic and hydrophobic antigens, and the systems’ biodegradability [107]. The safety of liposomes is evidenced by the number of clinically approved drugs in which they are incorporated. Challenges include the use of expensive materials, reproducibility issues, and the potential for oxidative degradation during storage and resultant instability [108,109,110]. In the late 1980s and early 90s, research into the use of liposomal vectors for the delivery of CS antigens culminated in multiple clinical trials. Early results with mice, rabbits, and monkeys demonstrated that antibodies specific to the CS protein (tetrapeptide B cell epitopes) were induced when it was encapsulated in dimyristoyl phosphatidylcholine/cholesterol (PC/Chol) liposomes with responses increasing upon incorporation of the adjuvant lipid A [62,63,64]. Though protection was not analyzed in these reports, a clinical trial using the liposome/antigen/lipid A system adjuvanted with alum was initiated based on the antibody results [12]. The IgG antibody response was greater than those obtained with alum in previous trials. At the highest doses sera inhibited hepatoma invasion by an average of 92%, potentially insufficient to prevent clinical symptoms developing. An anti-sporozoite vaccine needs to completely prevent hepatocyte infection to halt disease progression as any breakthrough infections can lead to blood stage infection. This demonstrates the benefits of having a vaccine that can target both the sporozoite and liver stages of malaria and the reasoning behind multi-stage vaccine development.

Non-liposomal lipid based particles have engendered little interest as malaria vaccine carriers. Moon *et al.* reported the use of interbilayer-crosslinked multilamellar vesicles (ICMVs) with a *Plasmodium vivax* CSP antigen, VMP001 [71]. ICMVs are described as nano-sized multilayered lipid vesicles with covalent cross-links between layers [135]. 180 nm particles with antigen both encapsulated and anchored to particle membranes were synthesized and induced a 9-fold greater immune response than particles lacking surface bound antigen [71]. This emphasizes the importance of controlling particle properties, as minor changes can have unexpected effects. When adjuvanted with monophosphoryl lipid A (MPLA), the antibodies targeted multiple epitopes with higher avidity than those produced with soluble protein and produced a balanced T helper response. The particles collected in draining lymph nodes, and the authors concluded that the enhanced, long lived humoral response was due to increased germinal center formation in close proximity to these particle depots and increased CD4+ T cell expansion and differentiation to antigen specific T_fh_ cells. The authors obtained similar results against the same antigen with 290 nm lipid enveloped poly (lactic-co-glycolic acid) (PLGA) particles [76]. These studies incorporate multiple features, which mark particles as attractive vectors. The ability to control the type of response, to target multiple epitopes, and to induce a long-lived response are hallmarks of successful vaccine design against malaria. Furthermore, enhanced germinal center formation in multiple studies suggests that either the properties of the particles or the lipids composing them are responsible. This provides an avenue for the induction of immunological memory and plasma B cells upon injection, important in the development of a long-lived response.

#### 4.1.2. Polymeric Particles

The descriptor “polymeric particles” encompasses a wide range of structures and conformations. These include natural and synthetic spheres (solid and hollow), nanogels, polymeric micelles, and dendrimers [7,136]. Among these candidates, only particles derived from polyesters and polyamino acids have seen in-depth exploration as malaria vectors, with the focus mainly on the polyesters, polylactic acid, and PLGA. Both solid and hollow particles ranging in size from about 100 nm to several microns have been used in drug and vaccine delivery [96,137]. Antigen/DNA can be encapsulated within the hollow core of a capsule, in the body of a sphere, or attached to the surface of either. A combination of the two is usually present [138]. PLGA is commonly used because both its monomers; lactic acid and glycolic acid, are biodegradable and easily dealt with by the body [111]. Its limited toxicity is further evidenced by incorporation in products approved by the FDA [138]. Targeting ligands can be attached to the surface to enable improved cellular binding and internalization [139]. For example, mannan has been covalently linked to PLGA particles to target C-type lectin receptors (CLRs) on DCs [140]. Other advantages include the ability to prolong the release of antigen and the co-encapsulation of adjuvants [112,113]. A potential problem is the loss of antigen immunogenicity either during synthesis or in the acidic environment formed upon degradation [110,114]. There have also been issues with scale-up and burst releases; large losses of antigen resulting from weak surface adsorption [141]. Thomasin *et al.* used a B cell CS epitope with universal T helper epitopes from tetanus toxins to synthesize a synthetic antigen, P30B2 [13]. This was loaded in PLGA microparticles with varying molecular weights and lactic: glycolic acid ratios. These properties allowed for control of the rate of antibody production via degradation, with overall titers longer lasting and of similar strength to incomplete Freund’s adjuvant (IFA) for all formulations. The same authors later found that combining microspheres of three different compositions and sizes induced a stronger antibody response to synthetic peptides than any singular particle, with said response similar in intensity and subclass distribution to peptides adjuvanted with IFA [73]. 

Extremely strong cytotoxic T cell responses are required to combat sporozoite infected hepatocytes and as such a number of studies have incorporated mixtures of B cell and CD8+ T cell epitopes in hopes of eliciting greater overall protection. Men *et al.* (1997) designed a system using six repeats of the NANP *P. falciparum* B cell epitope linked to universal tetanus toxin T helper epitopes and the cytotoxic epitope Pb9 in hopes of inducing cytotoxic T cell responses [74]. Chromate assays were used to demonstrate that the CD8+ T cell response was equivalent regardless of whether the peptides were encapsulated or injected with free particles. This suggested that the presence of the polymer was enough to improve MHC class I presentation. Using the same *P. berghei* epitope conjugated to 50 nm non-inflammatory polystyrene nanoparticles, Wilson *et al.* induced CD8+ T cell responses comparable to those obtained using montanide [77]. These responses were not developed when the antigen was not directly conjugated to the particle prompting the author to suggest that *in vivo* delivery of the peptide is vital in immune response development.

#### 4.1.3. Self-Assembling Protein Nanoparticles (SAPNs)

The relatively novel Self-Assembling Protein Nanoparticles (SAPNs) are composed of peptide chains deliberately designed to self-assemble in the right environment to form particles of a certain size and shape [116]. Similar to virus-like particles, they have the ability to repetitively present antigen in an ordered array [142]. This method of display has been linked to increased antibody affinity and suggests potential for strong immunogenicity [143]. Large scale production is possible but no data could be found on the potential toxicity of the constructs [144]. Kaba *et al.* used an immunodominant B cell epitope from CS to create 25 nm particles [78]. These were able to protect 60% of mice for six months upon initial challenge or 15 months upon secondary challenge when administered in both saline and montanide incomplete Seppic adjuvant 720 (ISA-720). This protection was associated with strong antibody titers, and using serum transfer was shown to be antibody dependent. It was further demonstrated using strains of mice deficient in CD4+ T cells that these cells were necessary for protection. The authors claimed that a sequence in the peptide was aiding DC maturation and enabling T cell activation, rendering adjuvants unnecessary. Later work by this group involved incorporation of PfCSP and PvCSP B and T cell epitopes into their SAPN [79]. A total of 90%–100% of mice were protected with just B cell epitopes incorporated into the SAPN constructs. After a year, 50% of mice were still protected. Using knockout mice and serum/splenocyte transfer the authors demonstrated that partial protection was obtained via both antibody mediated and CD8+ T cell mediated responses. This proved that both components of the system acted in concert to protect the animals. The authors later worked to define the mechanisms by which these responses developed [80]. They demonstrated inhibition of both sporozoite motility and hepatocyte invasion and growth, and linked this to sporozoite lysis via the classical pathway of complement activation. It was further shown that SAPNs are localized with TAP for prolonged periods of time in early endosomes, which is indicative of antigen cross presentation.

#### 4.1.4. Inorganic Particles

Inorganic particles have been used in a limited number of studies. This is generally a result of their lack of degradability and potentially toxic material components. Calcium carbonate is one such material. It has limited biodegradability and was incorporated with the polymers poly-l-lysine and poly-l-glutamic acid to form microparticle constructs loaded with a CS peptide containing a B and two T cell epitopes [81]. They demonstrated that a mixed antibody and cellular response developed, and that cellular immunity alone could not provide protection. The latter is a relatively common theme in the reviewed projects and is evidence that the immune responses against malaria vaccines are rarely simple with the concurrent development of humoral and cellular responses preferable. The differences between these studies limit the conclusions that can be drawn, but it is clear that particles do alter the immune response. The mechanisms by which each does so may differ depending on the particles properties but further investigation is needed to discover how.

### 4.2. Blood Stage Vaccines

It is thought that a good blood stage vaccine requires strong antibody production along with high levels of IFN-γ production to promote macrophage removal of infected cells [28]. A range of different particles have been used to meet these requirements and the most interesting are examined here.

#### 4.2.1. Lipid Based Particles

Sharma *et al.* entrapped soluble blood stage antigens inside both PC liposomes and those composed of lipids extracted from *E. coli* [84]*.* In contrast to the conventional liposomes, those developed from *E. coli* elicited CD8+ T cell production with 45% specific lysis of target cells compared to 2% with the PC liposomes. This was credited to the ability of the *E. coli* lipids to fuse with APCs and deliver antigens directly into the cytosol. These liposomes also induced greater CD4+ T cell responses with strong IL-2 and IFN-γ up-regulation and enhanced antibody titers. The importance of these responses was further evident in the superior survival rates (70% *vs.* 30%) of the mice following challenge. The authors obtained similar results when they compared liposomes synthesized using lipids from edible yeast *Saccharomyces cerevisiae* and PC liposomes, encapsulating the same antigens used previously [85]. This indicated development of both Th1 and Th2 helper cells which led the authors to conclude that both are involved in the control of the blood stage infection. The enhanced protection evident in these studies almost certainly results from increased antibody and Th1 development, though this may be aided by CD8+ T cell proliferation and associated cytokine production.

#### 4.2.2. Immune Stimulating Complexes (ISCOMs)

Immune Stimulating Complexes (ISCOMs) are so named for their ability to stimulate both humoral and cellular immune responses. They are spherical cage-like structures, around 40 nm in diameter, which form spontaneously from mixtures of saponin, cholesterol, and phospholipid [118]. Encapsulation has generally been limited to antigens which display amphipathic behavior, though this requirement has been relaxed somewhat with the development of ISCOMATRIX™ [145,146]. ISCOMATRIX™ differs from ISCOMs in that antigen is not incorporated during the synthesis of the structure but added afterwards to create the vaccine formulation [120]. Its successful use has also demonstrated the reproducibility and scalability of the technology. Human trials of saponin-based vaccines have seen them well tolerated [119]. Chopra *et al.* encapsulated two different ring-infected erythrocyte surface antigen (RESA) peptides by conjugating them to palmitic acid, creating an amphipathic molecule [91]. Both peptides elicited significantly (*p* < 0.001) higher IgG titers than alum adjuvanted antigen in the four strains of mice tested. Th1 biased responses were favored based on strong IgG2a and IgG2b levels with the authors suggesting that the bias towards these kinds of responses could be controlled by peptide choice. 

#### 4.2.3. Inorganic Particles

The only magnetic nanoparticles investigated as vaccine vectors are superparamagnetic iron oxide nanoparticles (SPIONs). These particles range from 1–30 nm and, as with many inorganic nanoparticles, exhibit unusual properties which make them attractive for different applications [147]. SPIONs have a single magnetic domain and can therefore be exposed to a magnetic field without remanence [148]. This has led to their use in magnetic targeting. For example, they have been magnetically concentrated in tumors and used as MRI contrast agents, situations in which organic particles are unsuitable [149,150]. Contrast agents based on SPIONs have been approved by the FDA, evidence of both their large scale capabilities and acceptable safety profile [121]. In contrast to most inorganic particles, they are biodegradable and readily excreted from the body [122]. Aggregation and stability problems are common in solution, though surface functionalization has mitigated this to some degree. This can take the form of polymeric or inorganic coating and/or ligand attachment (also allowing for *in vivo* targeting) [151]. Pusic *et al.* conjugated a recombinant MSP1-42 fragment to carboxyl groups on the outside of SPIONs with an amphiphilic polymer coating [14]. The size quoted was less than 20 nm, though the method of measurement was not clear and consequently is difficult to correlate with other studies. Intraperitoneal and intramuscular administration elicited responses from a greater percentage of mice than subcutaneous injection and sera from immunized mice inhibited parasite growth to a greater degree. Inhibition also exceeded that induced via protein administration with Freund’s adjuvant (FA) or ISA-51, potentially due to preferential induction of antibodies specific for inhibitory epitopes. Efficient uptake of the particles (without antigen) by APCs was demonstrated *in vitro* with expression of pro-inflammatory cytokines (TNF-α, IL-6) enhanced in DCs but not macrophages. In a similar study with quantum dots, the authors found that antibodies elicited against the conjugated peptide were significantly greater than those with FA or ISA-51 [94]. A more balanced Th1/Th2 response was obtained with the quantum dots with the response against SPIONs Th2 biased. Once again this highlights the importance of particle properties, in this case the material of the carrier, in determining the response against conjugated peptide. It should be noted that quantum dots contain heavy metals such as cadmium and zinc which are toxic to the human body [123,124]. This combined with their lack of biodegradability means that they can be only be considered as a model for nanoparticles in vaccine use. 

#### 4.2.4. DNA Vaccine Carriers

Few studies have used particles as vectors for malarial DNA vaccines. This is often due to difficulties encountered with encapsulation and degradation [152]. Al-Deen *et al.* electrostatically conjugated a plasmid vaccine based on the PyMSP1_19_ DNA fragment to the surface of polyetheleneimine (PEI) coated SPIONs (150 nm) via electrostatic interaction [92]. Intraperitoneal injection induced significantly (*p* < 0.001) greater antibody levels than intramuscular, intradermal and subcutaneous injection. This was attributed to improved access to APCs in and around the peritoneum and the containment of particles at the injection site through use of an external magnet. A strong IgG2a response was generated which correlates with a Th1 biased response. In other work with DNA vaccines Shuaibu *et al.* designed a layered PEI/γ-polyglutamic acid (γ-PGA) nanoparticle complex [117]. Positively charged PEI complexes with DNA and was then coated with negatively charged γ-PGA. The nanoparticles were less than 100 nm in size, with a zeta potential of −28 mV. Following intravenous administration, between 33% and 60% of mice survived challenge with infected red blood cells, with no survival in groups immunized with naked DNA [86]. IgG1 and IgG2b dominated the antibody response and this along with increased IL-4 production indicated a Th2 biased response. Following this they investigated the use of intraperitoneal injection which completely protected mice upon challenge [87]. Significantly greater IL-12p40, IFN-γ, IL-4 and IL-10 levels suggested a more balanced response than was obtained in the previous study, though this was also the case for intravenously administered particles (which also demonstrated improved survival). The authors suggest the improved survival rates may be a result of an improved DNA expression vector. Using an alternative target, the PyG18p transimidase related protein, similar results were obtained [88]. The authors point to previous work to conclude that effective T cell immunity was the most important correlate for protection against this antigen. In addition they demonstrated that particles without antigen were able to stimulate cytokine production, emphasizing the importance of understanding particle interactions with the body.

As expected the majority of the studies outlined here induced immune responses comparable to traditional adjuvants, though few directly studied how these antibody levels correlated with protection. Different particles and vaccine formulations were able to induce very different helper T cell biases. While no clear conclusion can be drawn on the importance of Th1 and Th2 responses, it is likely that vectors inducing a balanced response are most favorable for use with blood stage vaccine candidates. Once again this highlights one of the most attractive features of particle based vectors, their ability to control the type of immune response developed.

### 4.3. Multi-Stage Vaccines

A number of vaccine candidates incorporating epitopes from proteins displayed at different stages of the malarial life cycle have been developed in an effort to account for the extreme variability present in some alleles and provide more well-rounded protection. The synthetic *Plasmodium falciparum* polypeptide vaccine, SPf66 is one such candidate which has undergone clinical testing [153]. It is a polypeptide composed of three blood stage antigens linked via CS protein sequences. Rosas *et al.* encapsulated this peptide in PLGA in an attempt to improve its immunogenicity [96,98]. Particles of 1.2 µm were developed with peptide both encapsulated and associated with the surface. The IgG titers following subcutaneous injection were higher than those obtained with alum and comparable to FA, but the protection elicited was minimal. In later work by this group, administration method was analyzed in depth [100]. Intradermal injection of PLGA microparticles encapsulating SPf66 induced significantly (*p* < 0.05) greater antibody titers against SPf66 than subcutaneous injection. High levels of IFN-γ secretion indicated that intradermal injection resulted in a greater Th1 bias than subcutaneous or alum adjuvanted administration especially in the lymph nodes. This was credited to more efficient immune organ targeting as evidenced by significantly higher splenocyte proliferation. These results were reinforced using novel MSP2 peptides and the S3 short synthetic peptide containing epitopes from both sporozoite and blood stages, where intradermal administration induced greater antibody titers than subcutaneous injection and adjuvantation with montanide ISA-720 [101,102]. It was speculated that the intradermal response was higher due to preferential DC *vs.* macrophage uptake [154]. Most recently, alginate and alginate modified with the amino acid sequence arginine-glycine-aspartate (RGD, involved in cell adhesion) have been incorporated into PLGA particles [103]. These 1 µm sized particles incorporated the antigens SPf66 and S3 more efficiently with reduced surface adsorption than PLGA. In both cases greater antibody titers were seen. This was stated to be a result of alginate enhancing inflammatory cell migration and activation at the injection site. Alginate inclusion led to at least twofold increases in IFN-γ secretion and lymphocyte proliferation with RGD further increasing both 1.4 fold. This along with increasing IgG2a levels implied that the additives were promoting a more balanced CD4+ T cell response. The authors claimed that alginate was known to increase IFN-γ levels and that RGD likely enhanced CD4+ T cell uptake. 

### 4.4. Sexual Stage Vaccines

Very few studies have combined particulate vectors with malaria antigens from the sexual stage. This may in part be due to the difficulty inherent in testing their efficacy. While induction of antibodies is crucial for this stage of protection, it can be difficult to correlate antibody levels with the vaccines capability for inhibiting the spread of malaria. For instance even the most commonly used method of analyzing transmission blocking capability, the standard membrane feeding assay, only measures the oocyst burden. An understanding of how this relates to sporozoite numbers and overall disease transmission is still not clear, limiting its effectiveness [155]. Bhat *et al.* encapsulated a Pvs24 peptide in PLGA microparticles and delivered them intranasally and intramuscularly [105]. Intranasal delivery induced stronger antibody responses, especially when co-delivered with CpG oligodeoxynucleotide adjuvants, which trigger B-cell proliferation. Use of the sera in a mosquito membrane-feeding assay found a significant decrease in the number of oocysts present. The use of gel-core liposomes with Pfs25 antigen also produced antibody titers slightly better than those obtained with alum [106].

## 5. Connecting Nanotechnology and Malaria Vaccines

**Figure 3:**
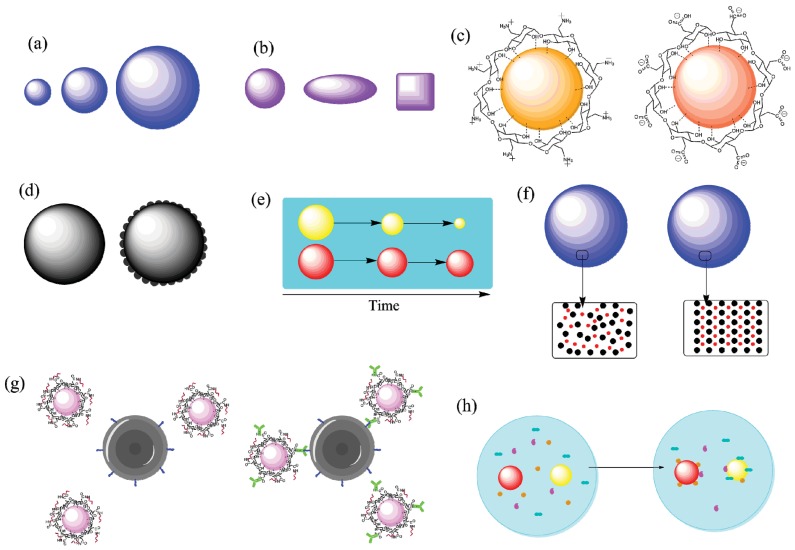
Properties of nanoparticles that can be controlled via synthetic or post-synthetic modification. (**a**) Size; (**b**) Shape; (**c**) Surface charge; (**d**) Surface roughness; (**e**) Biodegradation rate; (**f**) Crystallinity; (**g**) Active targeting; (**h**) Protein corona formation. Figure developed using ChemBioDraw Ultra 14.

The design of nanoparticles for use as malaria vaccine vectors should be based on an understanding of nanoparticle properties, the desired immune response and how these interact. The importance of carrier properties has only recently become clear. This was evident in the analysis undertaken in the previous section, with few studies giving more than a brief overview of particle characteristics (e.g., some studies summarized in Table 1). It is recommended that future studies utilizing carriers describe particle characteristics in depth, as these can be vital in understanding the reported results. Properties that can be controlled include size, shape, surface charge, hydrophobicity, material, hardness, method of antigen incorporation, crystallinity and surface roughness, and functionality [156,157,158]. A number of these are highlighted in Figure 3. There is no clear consensus on how each property affects the immune response, though a number of them have been studied. The lack of consistency within and the number of uncontrolled variables between these studies often leads to conflicting results. Nevertheless, an understanding of the particle and its properties is crucial to the intelligent design of a malaria vaccine system. This section summarizes and correlates some of the studies on particle characteristics with respect to vaccines in general, and notes how they may be beneficial in future particle-based malaria vaccine research.

### 5.1. Size

In the previous section, the size is given in a number of cases but it is often unclear which size is referred to. The most important size is that seen by the body: the hydrodynamic size. The hydrodynamic size refers to the size of a hard sphere, which diffuses in the same manner as the particle being measured. Unlike sizes derived from dried samples, it accounts for particle solvation and the development of hydration layers. The hydrodynamic size of a particulate system has a strong effect on the particles passage within the body and its uptake by APCs [156]. The majority of nanoparticles can be synthesized in a size-controlled manner, though there are limits on the available range. For example, polymeric, iron oxide, gold, and silica nanoparticles with diameters less than 50 nm have been synthesized [159,160,161,162]. The wide range of monodisperse particles available for purchase currently is evidence of the ease of size control. Use of different reagents in different ratios and layered growth on seed particles are two of the techniques commonly used to limit size during synthesis [161,163]. Little consensus exists in the literature on the optimum size for a vaccine vector. Unlike drugs, where a small size is preferred as it allows improved permeation and better distribution in the body, particle size affects vaccine efficacy in multiple ways. Most importantly, size can determine where in the body the vector travels and consequently which APC populations it interacts with, how it is taken up and processed by cells, and which cell populations uptake favors [164,165,166,167]. These factors then affect the magnitude and type of immune response. It is difficult to disentangle how the delivery and cellular interaction factors mentioned above influence the immune response, and therefore most comparisons are solely based on the responses generated. This has lead different groups to conclude that both small and large particles are preferable [168]. The malarial pre-erythrocytic immune response initially develops in lymph nodes and later on in the liver while the blood stage response is generated in the spleen [37,169]. Therefore, the ability to deliver a vaccine directly to these sites might be beneficial as was first demonstrated by Fifis *et al.* using the model protein OVA [167]. In analyzing the immune response particles with sizes varying from 20–2000 nm were studied and it was concluded that conjugation of antigen to 40 nm particles was preferable as this conjugate induced high IFN-γ and antibody responses correlated with increased uptake by DCs inclusive of a subset which promotes cross presentation [167]. The same authors later showed a bias towards CD8+ T cell activation with 40–49 nm particles and a CD4+ response for 93–123 nm particles [170]. Particles that were 67 nm produced only weak CD4+ and CD8+ responses. One of the most common issues with malaria vaccines and vaccines in general is a lack of CD8+ T cell induction. This is visible in the RTS,S vaccine and in a number of other candidates undergoing clinical trials with conventional adjuvants [5]. Therefore the ability to focus the response against a malarial pre-erythrocytic antigen could be very valuable. In similar work looking at particle distribution, Reddy *et al.* used polypropylene sulphide nanoparticles of 20 nm, 45 nm, and 100 nm without antigen to demonstrate that 20 nm particles drained to local lymph nodes to the greatest degree, while 100 nm particles stayed at the injection site [171]. Similarly 74% of 40 nm liposomes injected subcutaneously were taken up by the lymphatics while larger particles remained almost solely at the injection site [172]. There are limited data comparing different sized particles but other studies using small nanoparticles have also seen localization in lymph nodes [173,174,175,176]. This work suggests that the cut-off is ~100 nm, with larger particles staying at the injection site. Lymph node targeting is advantageous as it exposes the vaccine conjugate system to different subsets of APCs at much higher concentrations than are present in the periphery. This can potentially lead to stronger immune responses.

Analyzing the correlations between size and immune response, differences in the literature can be seen, for example in the work of Li *et al.* and Gutierro *et al.* [177,178]. The latter suggests that 1000 nm PLGA particles encapsulating bovine serum albumin induce greater immune responses than 500 nm and 200 nm particles while the former measured higher humoral and cellular responses with 230 nm particles compared to 708 nm particles conjugated to OVA. This highlights the importance of factors such as the method of antigen loading, particle size dispersity, particle type, and the route of administration amongst others; all of which may affect the immune response developed [168]. In any case, the ability to travel directly to lymph nodes makes smaller particles (<100 nm) attractive, but very few studies have directly compared the immune response of these particles with larger particles *in vivo*. *In vitro* evidence suggested that the optimal size for cellular uptake is 50 nm, although these studies were done with fibroblast, ovarian cancer and brain tumor cell lines, not APCs [179,180]. It has been proposed that this size range represents the thermodynamic optimum for receptor-mediated endocytosis [181,182]. Given that 20–200 nm particles are thought to be ingested via the same mechanism in DCs, it is plausible that a similar trend would be seen [164]. It is clear that hydrodynamic size is an important variable in vector design and should be reported in all cases, however, this was lacking in the studies analyzed in the previous section.

### 5.2. Surface Charge

One of the most attractive features of particle-based vectors is their amenability to surface modification. This includes both coating and encapsulation of the particle, and reaction of surface functional groups to produce a coating with alternate properties [156]. Coating is mainly used for one of two reasons: to improve the stability of the particle in solution or to alter the properties of the surface and enhance/alter the immune response. Stability can be improved by increasing the repulsive force between particles, thereby reducing their tendency to aggregate. Both steric and electrostatic forces are involved in aggregation and affect stability [183]. Various mechanisms for improving the immune response have been investigated, including altering the surface charge, changing the hydrophobicity, and the attachment of targeting ligands [158]. To take advantage of the negative charge of cell membranes, positively charged particles have been designed towards improved uptake and subsequent immune presentation [184,185]. A common issue with studies of this kind is ensuring that the modification of surface charge does not alter the other properties of the system. For instance, Thomas *et al.* found that cationic PLGA induced stronger humoral and mucosal immune responses against entrapped hepatitis B surface antigen than unmodified particles but acknowledged that other variables such as size and entrapment efficiency were likely to have influenced the results [186]. Henriksen-Lacey *et al.* observed that cationic liposomes with a tuberculosis antigen induced greater Th1 and Th17 cytokine responses than those with a neutral charge, potentially due to enhanced antigen absorption and the ability of the cationic material to act as an adjuvant [187]. There are fewer studies which look at nanoparticles, with Villanueva *et al.* and Cho *et al.* both reporting greater uptake of cationic particles in cancer cells *in vitro* [188,189]. In contrast, Foged *et al.* found no difference in DC uptake between 100 nm cationic and anionic particles without antigen [190]. Larger 1000 nm cationic particles were taken up to a greater degree than equivalent anionic particles. Comparable DC uptake suggests that anionic particles may be better in the nano-size range as they will exhibit less non-specific cellular uptake. It should be noted that none of these studies analyzed the immune response *in vivo*. Lastly there is evidence that internalization pathways and the type of immune response differ based on particle charge, though further investigation is required [191,192]. This suggests that while there may be potential benefits to altering particle charge in a malaria vaccine formulation, the limited data in this area constrain recommendations. If the antigen in question is charged or DNA based, attachment to an oppositely charged particle could produce a more stable formulation, as was demonstrated with SPIONs, via prolonged presentation [92]. 

### 5.3. Targeting Ligands

Any vector to which antigen can be adsorbed or conjugated can similarly be altered with targeting ligands. These ligands enable direct interactions with APCs and consequently they can improve cellular uptake and activation. Pattern recognition receptors (PRRs) on the surface of DCs allow identification of pathogen associated molecular patterns (PAMPs). This receptor subset includes toll-like receptors (TLRs) involved in recognition of and response to pathogen invasion, CLRs which aid endocytosis and nucleotide oligomerization domain (NOD)-like receptors that regulate the immune response. Agonists for these receptors can be conjugated to the surface of vectors to induce maturation, control the nature of the immune response (through TLRs) and/or increase particle uptake and subsequent antigen presentation (through CLRs) [193]. In the majority of TLR work, the agonist has either been encapsulated in a particle or co-administered as an adjuvant [194]. This was seen in a number of the liposomal studies outlined in the previous section, with the incorporation of the TLR-4 agonist lipid A [62,68]. Contrastingly, a number of studies have looked at surface conjugation of CLR ligands [195]. Kwon *et al.* conjugated monoclonal DEC-205 antibodies to polymeric particles encapsulating OVA, inducing twice as many CD8+ T cells than particles with a control antibody conjugated [196]. Similar enhancements of both CD4+ and CD8+ mediated immunity have also been reported [197,198]. Other receptors which have been targeted include mannose and DC-SIGN. Thomann *et al* demonstrated equivalent vaccine efficacy with decreased quantities of a cytotoxic ErbB2 antigen and a TLR2 adjuvant using a mannose agonist, while Cruz *et al.* found that DC-SIGN targeting increased the uptake of PLGA nanoparticles containing a tetanus toxin peptide but not microparticles [199,200]. Using the same particles, the authors demonstrated greater T cell proliferation [201]. Targeting to DCs is mostly required in the periphery, as DC concentrations in lymph nodes are far greater. Drainage via size control is likely preferable as it removes the need for ligand attachment and prevents early antigen presentation which can induce self-tolerance [193]. On the other hand, engaging targeting as a means for enhancing antigen uptake by APC subsets is attractive. Targeted uptake of particles by cross-presenting DCs promoting enhanced CD8+ responses would be useful, for instance in the development of a vaccine against a liver-stage malaria antigen.

### 5.4. Protein Corona and Material Characteristics

In the previous section, it was evident that a wide range of materials have been used in particle vectors for malaria vaccines. It was also clear that the choice of said material affected the immune response, even when the other characteristics of the particles were similar. For instance, Pusic *et al.* used quantum dots and SPIONs of a similar size in the same manner, but were only able to induce a strong subcutaneous response with quantum dots [14,94]. The elemental constituents, surface roughness and configuration, crystallinity, and the method of antigen incorporation all potentially alter the particles interaction with the body. Upon injection, particles interact with tissues, fluids, and a wide range of cells. The formation of a protein corona is the most important of these interactions. Serum and plasma contain an array of different proteins, each of which has the potential to adsorb to the surface of a particle [202]. The affinity of this interaction will depend on the particles composition as well as a number of other characteristics (size, charge, hydrophobicity) [203]. The adsorption of fibrinogen and immunoglobulins is dominant on ultra-small SPIONs while apolipoprotein bound more strongly to solid lipid nanoparticles [204,205]. Results of this kind have been demonstrated with a range of particles composed of different materials [206]. Adsorption of a protein layer increases the hydrodynamic size, can potentially influence the structure of the protein, and control the particles’ interaction with cells [207,208]. Wang *et al.* revealed that caveolae-based endocytosis of polymeric nanoparticles was promoted by the presence of an albumin coating [209]. Furthermore, it has been suggested that the presence of known opsonins, such as complement and immunoglobulins in the protein corona could be beneficial in the design of vaccines [203]. While this is just speculation, an understanding of protein coronae development and structure is as important as that of size in the design of malaria vaccine vectors. The other factor that needs to be considered in the choice of material is biocompatibility. This includes both biodegradability and toxicity; requirements which tend to limit the applicability of inorganic materials as was evident in the previous section [210]. 

### 5.5. Method of Antigen Incorporation

The method by which the subunit or DNA vaccine is combined with the particle system affects the subsequent immune response. Figure 2 highlights how the different systems investigated incorporate antigen. Particles can be mixed with the vaccine as are traditional adjuvants, or the antigen can be adsorbed or covalently bound to the surface [211]. Adsorption relies upon attractive forces such as electrostatic and van der Waal interactions and is weaker than covalent bonding [212]. Antigenic epitopes can also be incorporated into the particle framework, as in the previously mentioned SAPNs [142]. Hollow and porous nanoparticles allow for encapsulation of antigen, which is typically added to the initial reagent mixture [62]. The release rate depends on the degradation of the particle *in vivo*. It is evident that the encapsulation method used will lead to differences in the degree of antigen protection, the swiftness and length of the immune response, and the intracellular processing of the antigen. It was previously mentioned that encapsulation combined with surface display induced nine-fold greater antibody titers against a *P. vivax* CSP antigen than encapsulation alone in ICMVs [71]. While many of the variables alluded to in this section have only seen limited study, their existence demonstrates the possibilities nanoparticles provide as vectors, especially when compared with traditional adjuvants.

## 6. Conclusions

It is readily apparent that particle-based vaccine vectors can induce immunity relevant to the development of protection against malaria. A direct comparison between different synthetic vectors is difficult, as each study incorporates distinct antigens and injection regimes. Immune responses against specific antigens can be directed through particle selection, although this is not a guarantee that the same bias will result when other antigens are used. Effective improvement of vectors has been demonstrated through enhancement of both humoral and cellular responses, and the control of the type of cellular response, though this would benefit from a stronger mechanistic understanding. Few in-depth reports of *in vivo* particle interaction mechanisms are provided in the studies analyzed. These types of studies are generally extremely complex but can offer in-depth insight into not just the particle being tested, but those with similar properties without conjecture. Vital characteristics including protein corona structure, and nanoparticle size and charge are often neglected. As was evident in the previous section, the importance of these factors cannot be underestimated. 

Exploration into the manipulation and control of immune responses via a wide range of particle properties is producing promising results, which should influence future studies. SAPN development is producing systems which induce high antibody titers with high avidity for their epitopes, a requirement of both anti-sporozoite and anti-blood stage vaccine constructs. ICMVs and polymeric particles have been able to induce long-lived memory cells while PEI-γPGA systems are promising transfection agents. Size-dependent cytotoxic T cell induction may enable effective targeting of infected hepatocytes, overcoming an issue common to malaria vaccine candidates. Future vectors can be designed to complement different antigens and potentially create a multi-stage vaccine. Furthermore, systems incorporating multiple epitopes or multilayered particles capable of multiple antigen releases are possibilities. The positive results induced using biodegradable materials with proven toxicity profiles suggest that further research into particulate vectors will be beneficial.
